# Study of mental health of students with severe intellectual disabilities

**DOI:** 10.1192/j.eurpsy.2026.11248

**Published:** 2026-07-31

**Authors:** A. Zakrepina, T. Butusova, L. Pronina, Y. Sidneva, P. Tarnovskaya

**Affiliations:** 1 psychoeducation, Federal State Budgetary Scientific Institution Institute of Correctional Pedagogics; 2 neurorehabilitation, Clinical and Research Institute of Emergency Pediatric Surgery and Trauma; 3 neuropsychiatry, N.N.Burdenko National Medical Research Institute of Neurosurgery, Moscow, Russian Federation

## Abstract

**Introduction:**

A study of the mental health of schoolchildren studying according to special adapted programs showed that they experience persistent difficulties in mastering cognitive material. Among the special learning conditions, individual methods, differentiated volume of assistance in explaining new material, and drug support for mental health are distinguished.

**Objectives:**

to study the mental capabilities and analyze the learning difficulties of 5th grade students with severe intellectual disabilities, as well as to determine the impact of drug support on academic performance at school.

**Methods:**

The study involved 78 5th grade students studying according to adapted educational programs at school and at home in educational organizations in Moscow and the Moscow region. Teachers were offered a questionnaire to identify information about the medical support of each student.

The following methods were used: observation, pedagogical and psychiatric testing, cluster analysis.

**Results:**

The results showed that with mental health support, children with severe intellectual disabilities who received both pedagogical and medical assistance cope with educational tasks more effectively.

In group 1 of students (36%) with a stable mental health status (without concomitant mental and neurological diseases), 2 clusters were identified that were variable in academic performance indicators, had differences in the amount of assistance from the teacher and parents; among them, some students (20%) could complete assignments independently, and some (16%) with minor assistance from an adult.

In group 2 (64%), which consisted of children with various mental and neurological diseases, 3 clusters were identified and a wide variety of academic performance indicators was noted, which was directly related to the amount and quality of assistance provided, including medical. Among them, the majority of students (38%) studied at school individually with occasional medical support; another part (16%) – individually and in small groups of 2 children, with constant medical support, and some children (10%) were educated at home with medical course support and comprehensive rehabilitation programs.

**Conclusions:**

The study showed that those students with severe intellectual disabilities who received help from a psychiatrist, a neurologist show higher results in mastering the school curriculum; drug support stimulates intellectual activity, activates speech, improves memory, attention, reduces motor excitability and thus affects the success and productivity of learning.

**Disclosure of Interest:**

None Declared

